# Protecting quantum correlations of the *XXZ* model by topological boundary conditions

**DOI:** 10.1038/s41598-018-37474-x

**Published:** 2019-01-31

**Authors:** Shi-Ping Zeng, Hai-Long Shi, Xu Zhou, Xiao-Hui Wang, Si-Yuan Liu, Ming-Liang Hu

**Affiliations:** 1 Institute of Modern Physics, Northwest University, Xi’an, 710127 China; 2 School of Physics, Northwest University, Xi’an, 710127 China; 3 Shaanxi Key Laboratory for Theoretical Physics Frontiers, Xi’an, 710127 China; 4 Institute of Physics, Chinese Academy of Sciences, Beijing, 100190 China; 5 School of Science, Xi’an University of Posts and Telecommunications, Xi’an, 710121 China; 6 State Key Laboratory of Magnetic Resonance and Atomic and Molecular Physics, Wuhan Institute of Physics and Mathematics, Chinese Academy of Sciences, Wuhan, 430071 China; 7 University of Chinese Academy of Sciences, Beijing, 100049 China

## Abstract

The differences between the *XXZ* model with topological and periodical boundary conditions were compared by studying their entanglement, quantum discord, and critical temperature above which the entanglement vanishes. It shows that the different boundary conditions mainly affect bipartite quantum correlations of the boundary spins rather than that of other spin pairs. The topological boundary spins can protect entanglement and discord against strong magnetic fields while the periodical boundary spins can protect them against nonuniform magnetic fields. Compared with the periodical *XXZ* model, the critical temperature is significantly improved for the topological *XXZ* model. The topological *XXZ* model also allows us to improve significantly its critical temperature by increasing the strength of magnetic field, which is not feasible for the periodical *XXZ* model. It is therefore more promising for preparing entangled states at high temperature in the topological *XXZ* model.

## Introduction

Integrable models provide exact solutions for understanding some non-trivial physical phenomena in statistical physics, quantum field theory and condensed matter physics^1–4^. In general, integrable models can be divided into two classes: one possesses *U*(1) symmetry and the other does not. Previously, how to solve a model without *U*(1) symmetry is viewed as a formidable problem since a “local vacuum state” is not obvious. Recently, the authors of ref.^5^ have developed a systematic method called “off-diagonal Bethe Ansatz” (ODBA) to deal with it by adding an inhomogeneous term to the *T* − *Q* relation. The *XXZ* model with topological boundary condition is a typical integrable model without *U*(1) symmetry. The topological boundary condition not only makes the *XXZ* model lose *U*(1) symmetry but also endows it with some pretty unique properties. For instance, after a Jordan-Wigner transformation, it describes a *p*-wave Josephson junction embedded in a spinless Luttinger liquid^6–8^. Besides, each particle with momentum *k* must lock a hole with momentum −*k* to form a virtual bound state and thus the excitations of the topological *XXZ* model display topological nature^9^. The topological boundary condition can also profoundly affect quantum entanglement for the ground state of the *XXZ* model.

Quantum information science, as a new and developing research field, provides a variety of quantum correlations and enables us to capture the quantum characters of different quantum states in a mathematically rigorous fashion^10–22^. Entanglement is the first notion of quantum correlation to be discovered in 1935^23,24^ and is used to describe the separability of a given quantum state. Nowadays, entanglement is widely accepted as a quantum resource as real as energy due to the rapid development of entanglement-based quantum technology, such as teleportation^25,26^, quantum cryptographic key distribution^27^, and quantum metrology^28,29^. Meanwhile, various entanglement measures were proposed in the past few decades. There are also other quantum correlation measures, e.g., the quantum discord which is defined by the differences between total correlation and classical correlation^14^.

The characteristics of quantum correlations for various integrable models were intensively studied^30–35^. The critical temperature *T*_*c*_ above which the thermal entanglement of the considered model vanishes was also introduced^36^. For example, Wang explored thermal entanglement of the *XY* model and found that *T*_*c*_ is independent of the strength of the transverse magnetic field *B*^31–33^. Further investigation shows that the critical temperature *T*_*c*_ may be enhanced when the external magnetic field is nonuniform^34^. In other words, it is possible to create entanglement in a high temperature environment under inhomogeneous magnetic field. Previous studies mainly focused on investigating entanglement of the integrable models with periodical boundary conditions^37–44^, while the role of other boundary conditions was usually neglected. With these considerations, we are prepared to expound how the topological boundary condition affects thermal entanglement and quantum discord in the *XXZ* model by comparing those with the case of periodical boundary condition.

This work is organized as follows. We introduce the *XXZ* model with periodical and topological conditions first. Then an analysis of the bipartite entanglement, bipartite discord, tripartite entanglement, and critical temperature for them are carried out. The differences between quantum correlations in the *XXZ* model with periodical and topological conditions are compared in detail, through which it was found that the topological boundary condition is beneficial for the protection of entanglement against thermal fluctuations in the considered model. Last, we summarize our main conclusions.

## Results

### The *XXZ* Model

The Hamiltonian of the three-qubit *XXZ* model with nonuniform external magnetic fields reads

1 $$H=\sum _{i=1}^{3}\,[J({\sigma }_{i}^{x}{\sigma }_{i+1}^{x}+{\sigma }_{i}^{y}{\sigma }_{i+1}^{y})+{J}_{z}{\sigma }_{i}^{z}{\sigma }_{i+1}^{z}]+(B+b){\sigma }_{1}^{z}+B{\sigma }_{2}^{z}+(B-b){\sigma }_{3}^{z},$$

where *σ*^*α*^ (*α* = *x*, *y*, *z*) are the Pauli operators. *J* and *J*_*z*_ represent strength of the spin coupling, and *B* controls strength of external magnetic field along the *z*-direction. The parameter *b* is introduced to describe the inhomogeneity of magnetic field.

**Figure 1:**
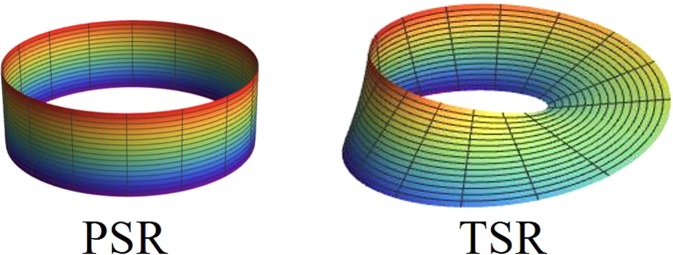
Two different boundary conditions. The periodical *XXZ* spin ring is denoted by PSR and the topological *XXZ* spin ring is denoted by TSR.

We consider two kinds of boundary conditions in this work, see Fig. 1. One is the periodical boundary condition (${\sigma }_{4}^{\alpha }={\sigma }_{1}^{\alpha }$, *α* = *x*, *y*, *z*), and the other is the topological boundary condition (${\sigma }_{4}^{\alpha }={\sigma }_{1}^{x}{\sigma }_{1}^{\alpha }{\sigma }_{1}^{x}$, *α* = *x*, *y*, *z*). For such a topological boundary condition, the spin on the last site connects with that on the first site after rotating a *π* angle along the *x* direction and thus forms a quantum Möbius strip in the spin space. This model is not only physically non-trivial due to its relevance to the realization of topological matter, but also non-trivial in solving due to its lack of the *U*(1) symmetry. By using the ODBA method, the exact solution of the topological *XXZ* model is obtained in ref. ^9^. However, the expressions of eigenstates given by ODBA method are complicated. Thus, we directly adopt the exact diagonalization method to obtain the energy spectrum (*E*_*i*_) and the eigenstates (|Ψ_*i*_〉) of the topological and the periodical *XXZ* model. The thermal equilibrium state of topological and periodical *XXZ* model can then be constructed by their energy spectrum and eigenstates as follows

2 $$\rho (T)=\frac{1}{Z}\,\sum _{i=1}^{8}\,{e}^{-\beta {E}_{i}}|{{\rm{\Psi }}}_{i}\rangle \langle {{\rm{\Psi }}}_{i}|,$$

where *β* = 1/*k*_B_*T*, *Z* = Tr(*e*^−*βH*^) is the partition function of the system, and the Boltzmann’s constant *k*_B_ will be set to 1 hereafter.

#### Quantum correlations of the *XXZ* model

We examine bipartite and tripartite correlations for thermal states of the three-qubit *XXZ* model, aimed at revealing effects of topological boundary condition on improving strength of the considered correlation and the corresponding critical temperature *T*_*c*_. The quantum correlations we considered are the bipartite entanglement quantified by concurrence *C*_*ij*_, the bipartite discord quantified by modified geometric discord *GD*_*ij*_, and the tripartite entanglement quantified by negativity. The subscript (*ij*) denotes the bipartite quantum correlations between the *i*-th spin and the *j*-th spin.

### Bipartite entanglement

The most basic entanglement measure is the entanglement of formation, which is intended to quantify the resources needed to create a given entangled state^45^. Concurrence is an entanglement-of-formation measure which applies for two-qubit states. It is defined as^46^

3 $$C(\rho )=\,{\rm{\max }}\,\{{\lambda }_{1}-{\lambda }_{2}-{\lambda }_{3}-{\lambda }_{4},0\},$$

where *λ*_*i*_ are square roots of the eigenvalues of the matrix $\rho \tilde{\rho }$ with ${\lambda }_{1}\geqslant {\lambda }_{2}\geqslant {\lambda }_{3}\geqslant {\lambda }_{4}$. $\tilde{\rho }=({\sigma }^{y}\otimes {\sigma }^{y}){\rho }^{\ast }({\sigma }^{y}\otimes {\sigma }^{y})$ and ρ^*^ is the complex conjugate of *ρ*. The concurrence *C* = 0 corresponds to an unentangled state while *C* = 1 corresponds to a maximally entangled state.

**Figure 2:**
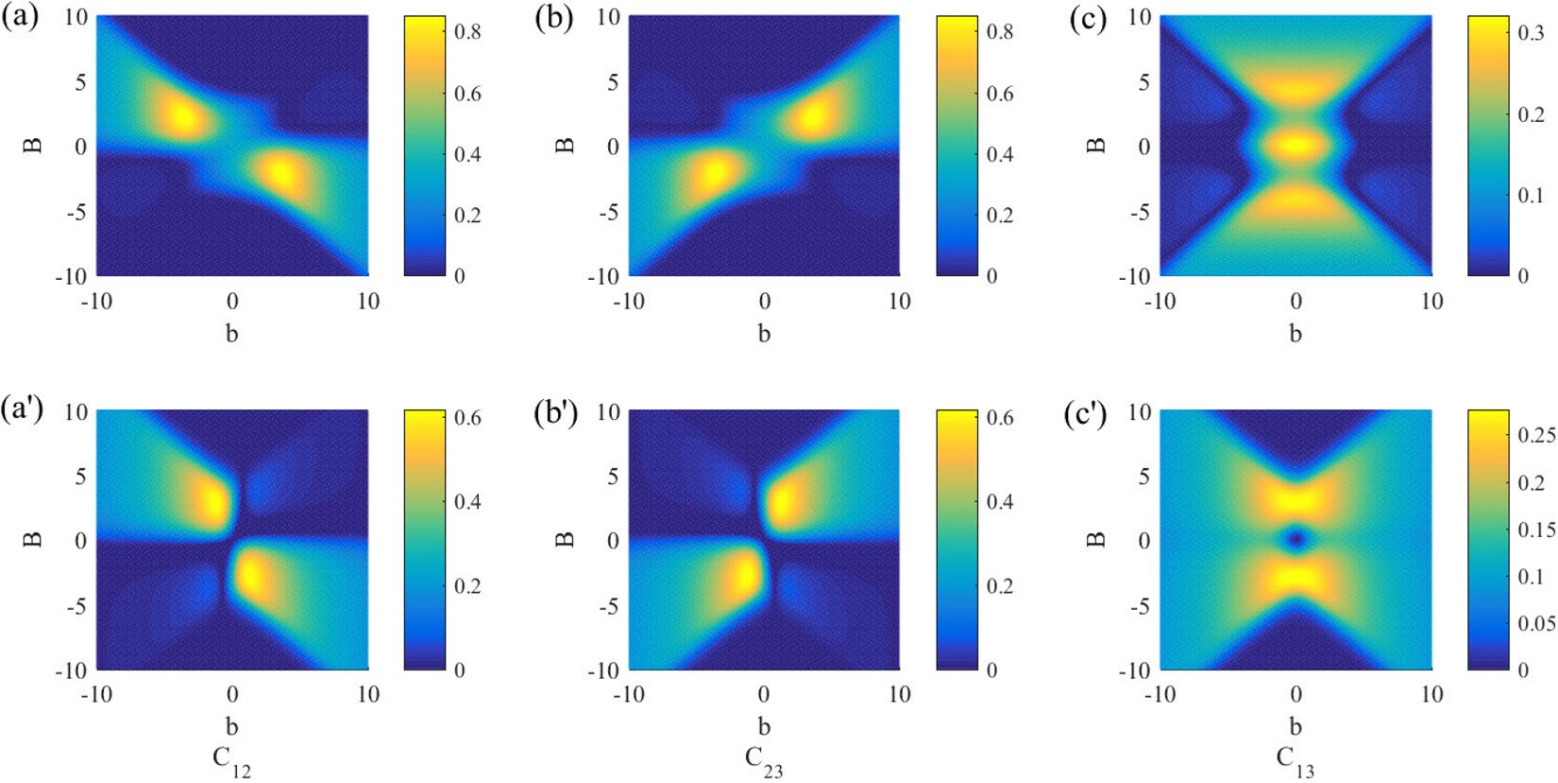
Concurrence versus *B* and *b* in the topological *XXZ* model and the periodical *XXZ* model with *T* = 1, *J* = 1 and *J*_*z*_ = 1.5. The maximum values of concurrence are 0.85, 0.85 and 0.32 for the topological case (**a**–**c**)] and 0.61, 0.61 and 0.28 for the periodical case (a′–c′).

Figure 2 shows dependence of *C*_12_, *C*_23_ and *C*_13_ on external magnetic fields *B* and *b*, from which one can note that the behaviors of *C*_12_ and *C*_23_ with different boundary conditions are similar. The boundary conditions only significantly affect entanglement between the boundary spins 1 and 3. For the periodical case, the concurrence for thermal states of the boundary spins can exist for a highly inhomogeneous magnetic field (large *b*) but it is easily to be destroyed by increasing the strength of magnetic field (large *B*), see Fig. 2(c′). For the topological case, it is just the opposite. Therefore, the differences induced by different boundary conditions mainly lie in the boundary spins, and we will concentrate on investigating behaviors of quantum correlations for them.

**Figure 3:**
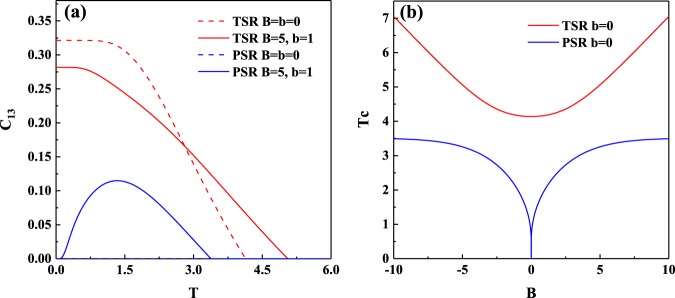
(**a**) Concurrence of the boundary spins *C*_13_ versus temperature *T* in the topological *XXZ* model and the periodical *XXZ* model with *J* = 1 and *J*_*z*_ = 1.5. The blue dash line coincide with horizontal axis. (**b**) Critical temperature *T*_*c*_ of concurrence *C*_13_ versus the strength of magnetic field *B* in the topological *XXZ* model and the periodical *XXZ* model with *J* = 1 and *J*_*z*_ = 1.5.

It is also worth noting that the maximal values of concurrence for the topological boundary condition case are always larger than those for the periodical boundary condition case. In particular, the boundary spins of the *XXZ* model with topological boundary condition possess bipartite entanglement even with *B* = 0, while this is not the case for that with the periodical boundary condition. These facts imply that a system with topological boundary condition may lead to more robust bipartite entanglement than a system with periodical boundary condition. The calculation of *C*_13_ versus *T* also support the above conjecture, e.g., from Fig. 3(a) one can found that the concurrence for the boundary spins with topological boundary condition is significantly larger than that with periodical boundary condition, whether it is at high temperature or low temperature.

The critical temperature *T*_*c*_ above which *C*_13_ vanishes was plotted in Fig. 3(b). It is clear that *T*_*c*_ for the case of topological boundary condition is higher than that for the case of periodical boundary condition. Moreover, *T*_*c*_ of *C*_13_ with topological boundary condition can be increased by increasing the strength of magnetic field *B*, which is not feasible for the periodical boundary condition case. In other words, by applying a sufficiently strong magnetic field *B*, the bipartite entanglement can be stored in the topological boundary spins at high temperature, whereas the periodical boundary spins do not have this advantage.

**Figure 4:**
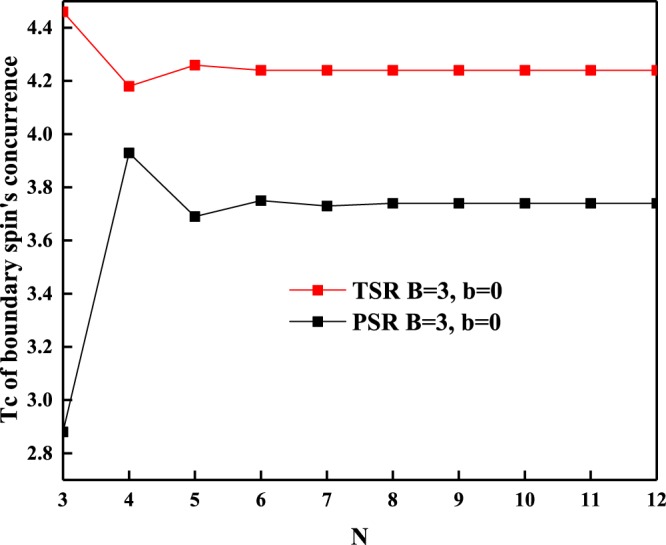
The scaling behavior of critical temperature *T*_*c*_ of boundary spin’s concurrence against the system size *N* with *J* = 1, *J*_*z*_ = 1.5, *B* = 3 and *b* = 0. The asymptotic value is 4.24 for TSR and 3.74 for PSR.

Figure 4 gives the scaling behavior of *T*_*c*_ of boundary spin’s concurrence versus the system size *N*, one can note that *T*_*c*_ of topological boundary spin’s concurrence is always higher than that of periodical case. Thus the topological boundary condition can protect bipartite entanglement against thermal fluctuations no matter how long the spin chain is.

### Bipartite quantum discord

Previous studies have shown that lots of quantum tasks could be realized successfully without entanglement. Then the quantum discord, which was defined as the differences between quantum mutual information and classical correlation, was introduced to measure the total amount of quantum correlations, thus it can characterize nonclassicality of correlations. The quantum discord defined in ref.^14^ is difficult to calculate even for general two-qubit states. In 2010, Dakić *et al*. proposed the geometric discord^47^, then the modified geometric discord was put forward where the density operator of the geometric discord was replaced by the square root of density operator^48^

4 $$GD({\rho }^{ab})=\mathop{{\rm{\min }}}\limits_{{{\rm{\Pi }}}^{a}}{\Vert \sqrt{{\rho }^{ab}}-{{\rm{\Pi }}}^{a}(\sqrt{{\rho }^{ab}})\Vert }^{2},$$

where the min is taken over all local von Neumann measurements ${{\rm{\Pi }}}^{a}=\{{{\rm{\Pi }}}_{k}^{a}\}$ on party *a*, ||·|| denotes the Hilbert-Schmidt norm, and

5 $${{\rm{\Pi }}}^{a}(\sqrt{{\rho }^{ab}}):\,=\sum _{k}\,({{\rm{\Pi }}}_{k}^{a}\otimes { {\mathcal I} }^{b})\sqrt{{\rho }^{ab}}({{\rm{\Pi }}}_{k}^{a}\otimes { {\mathcal I} }^{b}).$$

**Figure 5:**
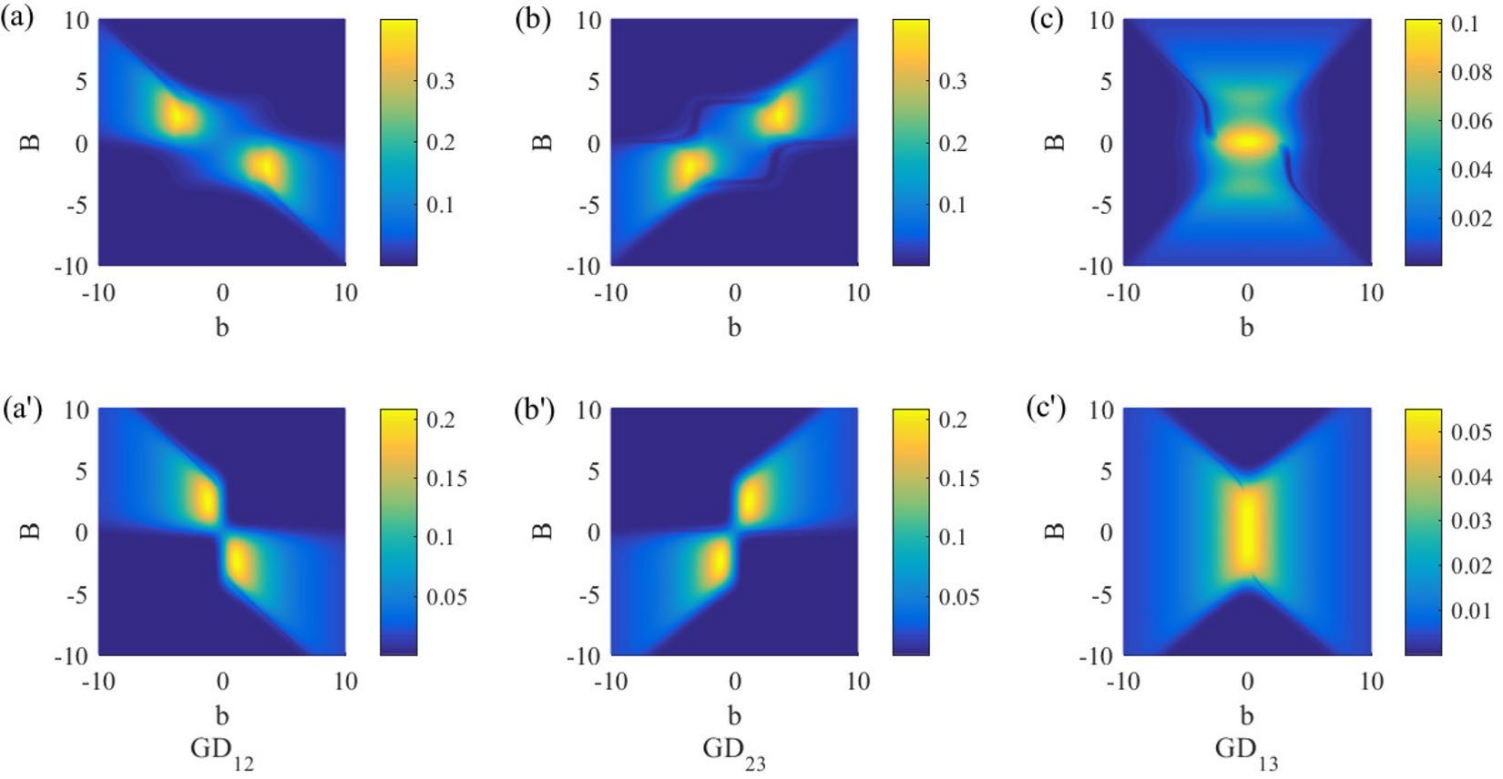
Modified geometric discord versus *B* and *b* in the topological *XXZ* model and the periodical *XXZ* model with *T* = 1, *J* = 1 and *J*_*z*_ = 1.5. The maximum values of discord are 0.32, 0.32 and 0.11 for the topological case (**a**–**c**)] and 0.16, 0.16 and 0.04 for the periodical case (a′–c′).

**Figure 6:**
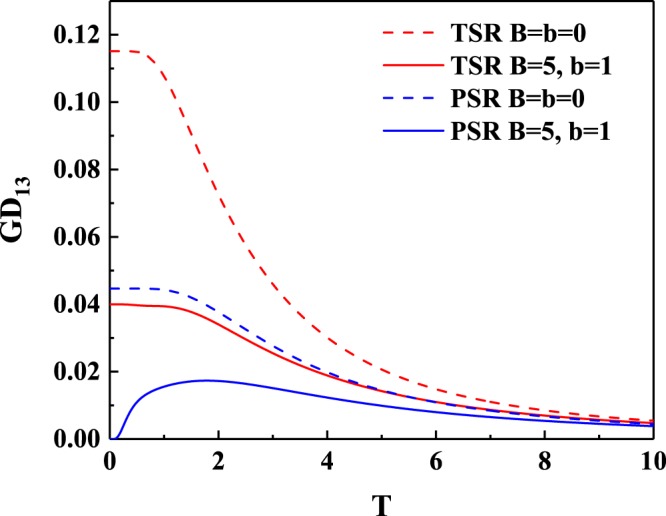
Modified geometric discord of the boundary spins *GD*_13_ versus temperature *T* in the topological *XXZ* model and the periodical *XXZ* model with *J* = 1 and *J*_*z*_ = 1.5.

Figure 5 shows modified geometric discord for different spin pairs of the *XXZ* model with periodical and topological boundary conditions, from which one can observe quantitatively the similar behaviors to those of bipartite entanglement: (1) the boundary spins are the main objects to bear the influence of different boundary conditions; (2) the topological boundary spins can store bipartite discord in the region of strong magnetic field *B*; (3) the periodical boundary spins can store bipartite discord in the region of strong inhomogeneous magnetic field *b*; (4) the maximal values of bipartite discord under topological boundary condition are larger than those under periodical boundary condition. Different from quantum entanglement, the quantum discord disappears only in the infinite temperature, and there does not exist a critical temperature *T*_*c*_. But the discord under topological boundary condition is always larger than that under the periodical boundary condition, see Fig. 6.

### Tripartite entanglement

Tripartite negativity^49^ is applied to measure tripartite entanglement in this work. It was defined as

6 $$N(\rho )=\sqrt[3]{({N}_{A-BC}{N}_{B-CA}{N}_{C-AB})},$$

where

7 $${N}_{i-jk}=\frac{1}{2}\,[\sum _{l}\,|{\lambda }_{l}({\rho }^{{T}_{i}})|\,-\,1],$$

and ${\lambda }_{l}({\rho }^{{T}_{i}})$ are eigenvalues of the partial transposed density martrix ${\rho }^{{T}_{i}}$ of the composite system with respect to subsystem *i*.

**Figure 7:**
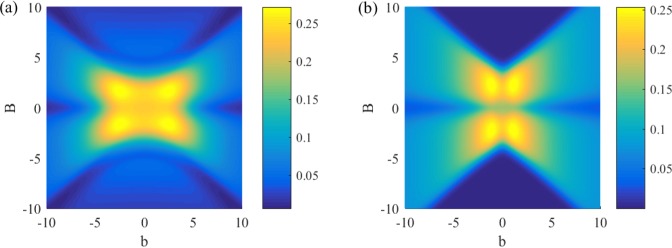
Negativity versus *B* and *b* in the topological *XXZ* model and the periodical *XXZ* model with *T* = 1, *J* = 1 and *J*_*z*_ = 1.5. The maximum value of negativity is 0.27 for the topological case (**a**) and 0.25 for the periodical case (**b**).

In Fig. 7, we showed dependence of negativity on *B* and *b*. There are two triangular regions representing strong magnetic fields *B* where the negativity vanishes for the case of periodical boundary condition while exists for the case of topological boundary condition. It means that the tripartite entanglement can exist in the region of strong magnetic field for the topological *XXZ* model.

**Figure 8:**
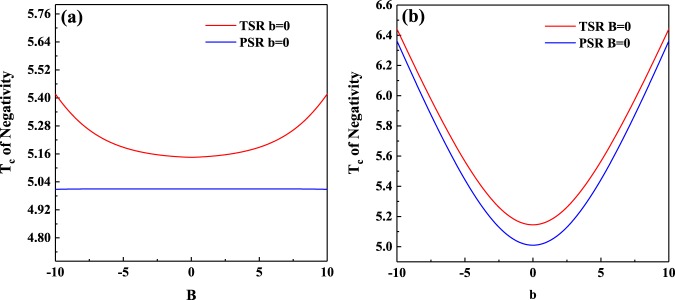
(**a**) Critical temperature *T*_*c*_ of negativity versus the strength of magnetic field *B* in the topological *XXZ* model and the periodical *XXZ* model with *J* = 1 and *J*_*z*_ = 1.5. (**b**) Critical temperature *T*_*c*_ of negativity versus the inhomogeneity of magnetic field *b* in the topological *XXZ* model and the periodical *XXZ* model with *J* = 1 and *J*_*z*_ = 1.5.

The previous researches^31–34^ show that the critical temperature *T*_*c*_ for the periodical *XY* model can be enhanced with a nonuniform magnetic field *b* but remains unchanged with increasing magnetic field *B*. For the periodical *XXZ* model, the same phenomenon is also found, see Fig. 8. It is unexpected that two completely different models show the similar properties. On the contrary, in addition to a nonuniform magnetic field, the topological boundary condition allows us to improve *T*_*c*_ for the *XXZ* model via another approach, i.e., increasing the strength of magnetic field *B*. There is another interesting phenomenon that *T*_*c*_ of tripartite entanglement is higher than *T*_*c*_ of bipartite entanglement in periodical *XXZ* model. Nevertheless, in the topological *XXZ* model, *T*_*c*_ of tripartite entanglement will lower than *T*_*c*_ of bipartite entanglement. This indicates that there is a region in the topological *XXZ* model where bipartite entanglement exists but tripartite entanglement vanishes. For the periodical *XXZ* model, there is a region in which the existence of tripartite entanglement is a necessary condition for existence of bipartite entanglement.

## Discussion

In this work, the three-qubit *XXZ* model with periodical and topological boundary conditions have been investigated by calculating their bipartite entanglement, bipartite discord, tripartite entanglement, and corresponding critical temperature. The results reveal that the different boundary conditions mainly affect bipartite quantum correlations of the boundary spins instead of other spin pairs. In the region of strong magnetic field *B*, the bipartite entanglement and bipartite discord can be stored in the topological boundary spins. On the contrary, the *XXZ* model with periodical boundary condition is conducive to storing bipartite entanglement and bipartite discord in a nonuniform magnetic field.

Generally speaking, the topological boundary condition protects entanglement against thermal fluctuations in the *XXZ* model because critical temperature of bipartite entanglement in the topological boundary spins is always higher than that in the periodical boundary spins, even when the system size *N* grows. Moreover, the critical temperatures of tripartite entanglement in the topological *XXZ* model are all higher than those in the periodical *XXZ* model. The topological boundary condition also allows us to improve critical temperature by the following two approaches: increasing the strength of magnetic field *B* or the inhomogeneity of magnetic field *b*. However, increasing the strength of magnetic field is useless for improving the critical temperature of the periodical *XXZ* model, especially for the case of tripartite entanglement. From this point, the periodical *XXZ* model is similar to the periodical *XY* model. Another interesting finding is that there is a region for the topological *XXZ* model where bipartite entanglement exists but tripartite entanglement vanishes.

Topological boundary condition not only breaks the *U*(1) symmetry making the *XXZ* model hard to solve but also protects the entanglement against thermal fluctuations. Besides, we can increase the strength of magnetic field to make entanglement alive at relatively high temperature, which is impossible for the periodical case. We believe that systems with topological boundary condition will exhibit their unique properties in entanglement-based quantum information technologies. In particular, it would be interesting to make a thorough calculation for the case of infinite topological *XXZ* chain via ODBA method.
